# Pressure ulcers in German hospitals: Analysis of reimbursement and length of stay

**DOI:** 10.1515/med-2023-0839

**Published:** 2024-02-13

**Authors:** Nils Lahmann, Martha Feh Mayer, John Posnett

**Affiliations:** Geriatrics Research Group, Charité Universitätsmedizin Berlin, Charitéplatz 1, 10117 Berlin, Germany; Medical School Berlin University, Rüdesheimer Str. 50, 14197 Berlin, Germany; Value and Access Strategy, IGES, Berlin, Germany; Independent Health Economics Consultant, York, UK

**Keywords:** Germany, pressure ulcer, hospital-acquired, length of stay, reimbursement, G-DRG

## Abstract

**Objective:**

Hospital-acquired pressure ulcers are an important indicator of the quality of care. Most pressure ulcers are avoidable with a robust protocol for prevention, but prevention activities often have a low priority for senior management because the true costs to the hospital are not visible. Our aim was to raise awareness of the value of pressure ulcer prevention by estimating the excess length of inpatient stay associated with hospital-acquired pressure ulcers, and by assessing whether additional costs are covered by increased reimbursement.

**Methods:**

National activity data for hospitals in Germany are available through the InEK Data Browser. Data were extracted covering discharges from German hospitals between January 1 and December 31, 2021. Cases were selected according to the presence of a pressure ulcer diagnosis using ICD-10-GM codes L89.0–L89.3. Information was extracted for the ten most common German Diagnosis-Related Group (G-DRG) codes in patients with a secondary pressure ulcer diagnosis on mean length of stay and average reimbursement. Ulcer-associated excess length of stay was estimated by comparing cases within the same G-DRG with and without a pressure ulcer diagnosis.

**Results:**

Mean length of stay was higher in patients with a pressure ulcer than in patients with no ulcer by between 1.9 (all ages) and 2.4 days (patients aged ≥65) per case. In patients aged ≥65 years, 22.1% of cases with a pressure ulcer had a length of stay above the norm for the DRG. In the German system length of stay above the norm is not normally reimbursed. Excess length of stay between 1.9 and 2.4 days leads to a potential cost to a hospital of between 1,633€ and 2,074€ per case.

**Conclusion:**

Hospital-acquired pressure ulcers represent an important source of cost for a hospital which highlights the potential value of effective prevention.

## 1 Introduction

The incidence of hospital-acquired pressure ulcers is an important indicator of the quality of care [1]. Pressure ulceration typically results from a sustained period of localised pressure which restricts blood-flow to the skin and subcutaneous tissues, leading ultimately to cell death. In severe cases pressure damage can lead to a deep wound extending through to bone [2]. Most pressure ulcers should be avoidable with a robust protocol for prevention [3], but prevention activities often have a low priority for senior management because the true costs to the hospital are not visible.

According to the IQTIG quality indicators report for 2020 [4], 21.6% of pressure ulcers treated in German hospitals were not present on admission, and this suggests that more than 82,000 of the 395,980 pressure ulcers treated in 2021 were hospital-acquired. In the 10 years between 2012 and 2021, an average of 743 patients died annually in Germany from complications of pressure ulceration [5]. Apart from the impact on patient welfare, pressure ulceration can also have an impact on the hospital because of the costs of delayed discharge, readmission and/or higher intensity of nursing care. The aim of this analysis was to raise awareness of the value of investments in pressure ulcer prevention by estimating the excess length of stay associated with a hospital-acquired pressure ulcer, and assessing whether additional bed-day costs are covered by increased reimbursement.

## 2 Materials and methods

### 2.1 Materials

Hospitals in Germany are obliged to provide activity data annually to the German Institute for the Reimbursement System in Hospitals (InEK GmbH), and these data are made available to researchers in anonymised form through the InEK Data Browser [6]. Our analysis was based on data extracted from the Data Browser in January 2023, covering inpatient cases discharged from German hospitals in the period January 1 to December 31, 2021. Cases are classified by primary and secondary diagnosis using the modified ICD-10-GM classification system [7] mapped to a unique German Diagnosis-Related Group (G-DRG) code.

Cases for analysis were selected according to the presence of a pressure ulcer diagnosis using ICD-10-GM codes L89.0–L89.3 corresponding to pressure ulcer stages 1–4, and code L89.9 assigned when the stage of the ulcer is not known (Table 1). Information was extracted for G-DRG codes in the analysis population on average (mean) length of inpatient stay, the proportion of cases with length of stay above or below the norm for the DRG and the fixed-rate reimbursement amount. InEK also provides information on episode costs from a sample of around 300 hospitals, and this cost collection forms the basis of the G-DRG fixed-rate payment. Reimbursement values for each G-DRG are available in the InEK Data Browser and the analysis uses reimbursement values for 2023.

**Table 1:** ICD-10 GM pressure ulcer diagnosis codes

ICD-10-GM pressure ulcer code	Definition	Brief description
L89.0	Decubitus, stage 1	Pressure zone with redness which cannot be pushed away with intact skin
L89.1	Decubitus, stage 2	Pressure ulcer with blister (serum-filled) (open) (ruptured). Partial loss of skin including epidermis and/or dermis
L89.2	Decubitus, stage 3	Pressure ulcer with loss of all skin layers with damage or necrosis of subcutaneous tissue that may extend to underlying fascia
L89.3	Decubitus, stage 4	Pressure ulcer with necrosis of muscles, bones or supporting structures (e.g., tendons or joint capsules)
L89.9	Decubitus, stage unspecified	Pressure ulcer without indication of stage

Source: www.dimdi.de/static/de/klassifikationen/icd/icd-10-gm/kode-suche/htmlgm2023/.

Information on the incidence of hospital-acquired pressure ulcers in Germany was derived from the literature and from a quality indicator report published by the Institut für Qualitätssicherung und Transparenz im Gesundheitswesen [4]. Average daily hospital costs per occupied bed-day in 2020 were sourced from AOK-Bundesverband [8]. The daily cost includes the full running costs (variable costs) but excludes costs for capital investment.

### 2.2 Methods

Our study is a form of cost analysis [9] which aims to estimate the opportunity cost to a hospital in Germany of patients who develop a pressure ulcer after admission. The perspective is acute general hospitals in Germany and the time horizon is from patient admission to discharge. Opportunity cost is estimated by first comparing inpatient length of stay of patients with a secondary pressure ulcer diagnosis with the length of stay of patients in the same G-DRG without an ulcer. The second part of the analysis compares reimbursement for the G-DRG code based on the primary diagnosis, with the opportunity cost to the hospital associated with the additional ulcer-associated length of stay. The opportunity cost is expressed in terms of annual ulcer-associated bed-days lost valued at (a) an average cost per occupied bed-day (864€) [8] or (b) in terms of the number of additional cases which could be treated if the number of incident pressure ulcers was reduced. Because InEK data are presented in aggregated form it is not possible to estimate confidence intervals around differences in length of stay or to test the statistical significance of observed differences. However, since the data represent the total population and not a sample, these inferential statistical measures are to be regarded as subordinate. Therefore, the means and median values can be interpreted directly.

In the G-DRG system, each patient is assigned a primary diagnosis at admission and potentially several secondary diagnoses at admission or throughout the inpatient stay. The InEK data do not distinguish between diagnosis codes assigned at admission or discharge, but they do distinguish between cases in which pressure ulcer was the primary diagnosis (and likely reason for admission) and cases in which pressure ulcer was a secondary diagnosis. A G-DRG code is assigned at discharge based on the complete patient episode. Our analysis estimates the difference in length of stay between cases with the same G-DRG code and no pressure ulcer, and cases with pressure ulcer as a secondary diagnosis.

- All the cases in the Data Browser where a pressure ulcer was coded either as a primary (*n* = 12,856) or secondary diagnosis (*n* = 383,124) were selected (including L89.9, stage unspecified).
- For cases where pressure ulcer was a secondary diagnosis, the ten most frequently occurring G-DRG codes were selected, ranked by the number of cases.
- For these G-DRG codes, average (mean) length of stay was extracted from the data and compared between cases with no pressure ulcer diagnosis and cases with a secondary pressure ulcer diagnosis, to estimate the ulcer-associated excess length of stay.

The per case reimbursement was estimated for each of the ten most frequent G-DRGs and compared with the cost per case calculated by combining the reimbursement with the additional cost associated with excess length of stay for cases with a secondary ulcer diagnosis.

In the German system, hospital reimbursement per case is determined by two factors: a flat-rate payment which depends on the G-DRG code, plus a daily amount to cover nursing costs which varies with length of stay. The flat-rate payment does not change if a patient develops a pressure ulcer after admission, except in cases where interventions to treat the ulcer involve a higher effort (higher complexity) than the primary diagnosis [10]. This will normally occur only if the incident ulcer is in the most severe category (stage 4), and because stage 4 ulcers represented less than 10% of cases with a secondary pressure ulcer diagnosis in Germany in 2021 this will be relatively rare (Table 2).

**Table 2:** Pressure ulcers in German hospitals, 2021

Stage of ulcer	Pressure ulcer as secondary diagnosis			Pressure ulcer as primary diagnosis			All pressure ulcer cases	
	*N*	%	Length of stay*	*N*	%	Length of stay*	*N*	%
Stage 1	64,654	16.9	19.43	49	0.4	9.05	64,703	16.3
Stage 2	207,872	54.3	21.46	737	5.7	10.39	208,609	52.7
Stage 3	77,181	20.1	22.15	3,362	26.2	15.1	80,543	20.3
Stage 4	28,319	7.4	23.73	8,617	67.0	20.40	36,936	9.3
Unspecified	5,098	1.3	18.41	91	0.7	9.6	5,189	1.3
								
Total	383,124	100.00		12,856	100.00		395,980	100.0
Mean			21.04			12.91		
Median			21.46			10.39		

Source: InEK Data Browser, 2021, *days.

The flat-rate payment is partly based on an assumed normal (average) length of stay for each G-DRG code. When length of stay is longer than the norm for the G-DRG a hospital incurs an additional cost if the average cost per bed-day exceeds the daily nursing time payment.

## 3 Results

**Figure 1:**
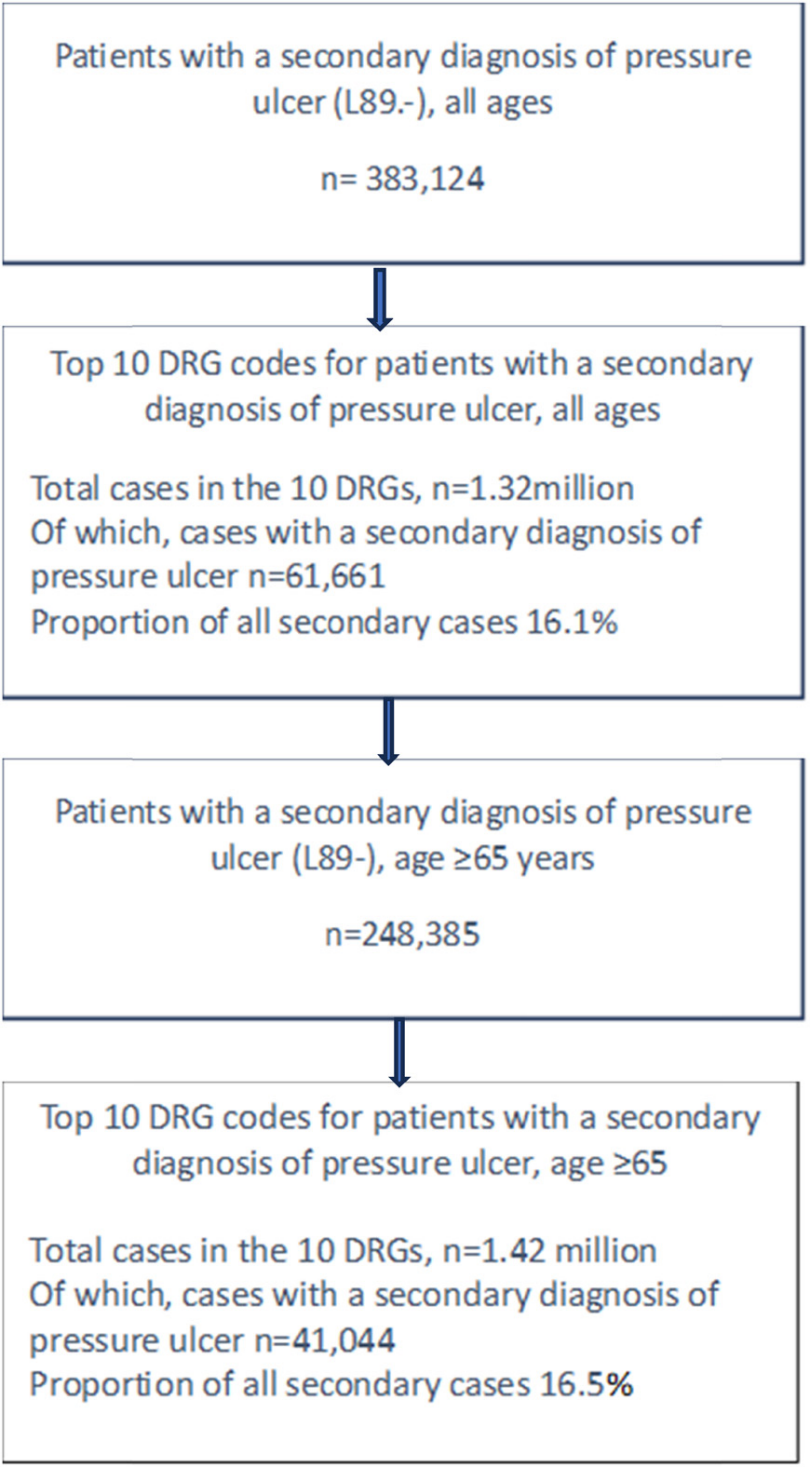
Flow diagram. Case numbers for the period January 1 to December 31, 2021.

In 2021, 395,980 cases were recorded in German hospitals of patients with at least one pressure ulcer. Most of these ulcers involved an open wound (stage 2 or higher) and more than 90% were cases where the ulcer was a secondary diagnosis (Table 2). In the analysis population 12,856 cases were recorded in German hospitals of patients with a primary diagnosis of pressure ulcer. Mean length of stay for these patients ranged from 9.1 to 20.4 days depending on the category of ulcer, with an overall mean of 12.9 days (median 10.4 days) (Table 2). The number of cases recorded with at least one secondary diagnosis of pressure ulcer was 383,124. Mean length of stay ranged from 18.4 to 23.7 days depending on the category of ulcer, with an overall mean of 21.0 days (median 21.5 days) (Table 2). Not all cases with a secondary pressure ulcer diagnosis were hospital-acquired, but the IQTIG Quality indicators report for 2020 [4] suggests that 21.6% of pressure ulcers in hospitalised patients were not present on admission. In 2021 the ten most frequent G-DRG codes for cases with a secondary pressure ulcer diagnosis (all ages) accounted for 1.32 million cases, of which 61,661 were cases with a secondary pressure ulcer diagnosis (Figure 1). Mean length of stay for cases without a pressure ulcer was 11.6 days (median 9.3 days) compared with a mean of 13.4 days (median 11.2 days) for cases with an ulcer, a difference of 1.9 days per case (Table 3). The mean number of cases exceeding the normal length of stay was 7.0% (median 6.6%). In cases with a pressure ulcer the number was 14.6% (median 14.8%), a difference of between 7.6 and 8.2 percentage points (Table 3).

**Table 3:** Top ten G-DRG codes, cases with a secondary diagnosis of pressure ulcer, all ages

DRG code	Short description	Average LOS	Average LOS with PU	Δ LOS with PU	Long stays	Long stays with PU	Δ Long stays with PU	No. of cases	No. of cases with PU
		Days	Days	Days	%	%		*N*	*N*
F62C	Heart failure and shock w/out severe CC	8.4	10.8	***+2.4***	6.75	15.92	***+9.17***	309,315	11,819
E79C	Infections and inflammations of respiratory organs	6.7	8.5	***+1.8***	6.39	14.26	***+7.87***	247,931	10,998
L63E	Infections of the urinary organs w/out extremely severe CC	5.7	7.6	***+1.9***	8.14	18.25	***+10.11***	147,107	7,179
I41Z	Geriatric early rehabilitation, complex treatment for diseases and disorders of the musculoskeletal system	19.8	21.1	***+1.3***	5.35	8.94	***+3.59***	68,244	6,866
K62C	Various metabolic diseases except para/tetraplegia w/out complicated diagnosis	5.2	7.2	***+2.0***	6.51	15.34	***+8.83***	140,994	6,168
G67B	Esophagitis, gastroenteritis, gastrointestinal haemorrhage, ulcer disease with complicating factors or extremely serious CC	4.0	6.5	***+2.5***	6.24	22.66	***+16.42***	265,218	3,884
I34Z	Geriatric early rehabilitation, complex treatment with specific OR procedure for diseases of the musculoskeletal system	23.7	25.7	***+2.0***	5.82	10.85	***+5.03***	42,840	3,871
E79A	Infections and inflammations of the respiratory organs with complex diagnosis or extremely severe CC	11.1	12.7	***+1.6***	6.81	10.72	***+3.91***	29,062	3,770
T60E	Sepsis without complicating constellation, except for condition after organ transplantation	10.2	11.6	***+1.4***	10.92	17.21	***+6.29***	31,709	3,648
F48Z	Geriatric early rehabilitation, complex treatment for diseases of the circulatory system	20.7	22.7	***+2.0***	6.76	11.71	***+4.95***	33,427	3,458
	Mean	11.6	13.4	***+1.9***	7.0	14.6	***+7.6***		
	Median	9.3	11.2	***+1.9***	6.6	14.8	***+8.2***		
	Total							1,315,847	61,661

Source: InEK Data Browser, 2021.

PU = pressure ulcer; LOS = length of stay; Long stays = percent of cases with length of stay longer than the norm for the DRG.

Bold values indicate to highlight points of interest.

The difference between reimbursement and nominal costs for the ten G-DRG codes is shown in Table 4. Total reimbursement per case at 2023 values is the flat-rate payment for the relevant DRG plus nursing cost reimbursement for the actual length of stay rounded to the nearest day. Nursing costs are reimbursed at a reference value of 230€ per day which varies between hospitals and according to the G-DRG code [11]. The mean reimbursement was 6,153€ (median 4,756€) per case (range 2,485–14,974€). The opportunity cost to the hospital was calculated by multiplying the excess ulcer-associated length of stay by an estimate of the average cost per occupied bed-day in German hospitals (864€) [8]. The difference between reimbursement and the estimated cost for patients with pressure ulceration was 1,633€ (median 1,649€) per case (Table 4).

**Table 4:** Top ten G-DRG codes, cases with a secondary diagnosis of pressure ulcer; average reimbursement, cost and excess cost per case, all ages

DRG code	Fixed rate DRG reimbursement* (€)	Nursing reimbursement per day** (€)	Total case reimbursement at rounded average LOS (€)	Cost at rounded excess LOS*** (€)	Excess cost per case (€)
F62C	2,700	180.55	*4144.40*	6218.00	***2073.60***
E79C	2,420	227.01	*4009.07*	5564.27	***1555.20***
L63E	1,900	185.22	*3011.31*	4652.91	***1641.60***
I41Z	5,476	158.19	*8639.88*	9763.08	***1123.20***
K62C	1,944	199.53	*2941.63*	4669.63	***1728.00***
G67B	1,752	183.33	*2485.33*	4645.33	***2160.00***
I34Z	10,552	180.09	*14974.16*	16602.16	***1728.00***
E79A	4,132	236.69	*6735.62*	8118.02	***1382.40***
T60E	3,248	211.99	*5367.91*	6577.51	***1209.60***
F48Z	5,856	165.07	*9322.49*	11050.49	***1728.00***
Mean			*6153.18*	7786.14	***1632.96***
Median			*4756.16*	6404.96	***1648.80***

*Calculated using the 2023 federal base value (4,000€). Case values may differ slightly depending on the region. **Calculated using the 2023 federal nursing base value of 230€. Case values may differ between hospitals. ***Calculated at an average 2020 value of 864€ per additional pressure ulcer-associated bed-day (Table 3).

Bold values indicate to highlight points of interest.

According to the IQTIG quality report [4], more than 80% of stage 2–4 hospital-acquired ulcers occurred in patients aged 60 years or more and 45% occurred in patients aged at least 80 years (Table 5). Because a large proportion of hospital-acquired pressure ulcers occur in the elderly population the analysis of InEK data was repeated for a population aged ≥65 years. The ten most frequent G-DRG codes in the InEK population aged ≥65 years accounted for 1.42 million cases, of which 41,044 had a secondary pressure ulcer diagnosis (Figure 1). The mean length of stay for cases without a pressure ulcer was 5.4 days (median 5.0 days) compared with a mean of 7.8 days (median 7.3 days) for cases with pressure ulcer as a secondary diagnosis, a difference of 2.4 days per case. The number of cases exceeding the normal length of stay for the G-DRG was 7.1% (median 6.9%), compared with a mean of 22.1% (median 18.6%) for cases with an ulcer, a difference of between 11.7 and 15.0 percentage points. The mean reimbursement was 3,302€ (median 2,809€) per case (range 1,992–7,294€). The difference between reimbursement and the estimated cost for patients with pressure ulceration was 2,074€ (median 1.814€) per case.

**Table 5:** Patients treated in German hospitals with at least one hospital-acquired pressure ulcer stage 2–4, by age (2020)

Age distribution	Number of patients with at least one hospital-acquired pressure ulcer stage 2–4	%
20–59 years	6,784	11.35
60–69 years	10,108	16.90
70–79 years	16,141	26.99
≥80 years	26,764	44.76
Total*	59,797	100.0

Source: IQTIG, 2021.

*Total refers to stage 2–4 only, and is based on the IQTIG sample population.

## 4 Discussion

In each of the ten G-DRGs, average length of stay was higher in patients with a pressure ulcer than in patients with no ulcer by between 1.9 (all ages) and 2.4 days (age ≥65) per case. In patients aged ≥65, 22.1% of cases had a length of stay above the norm for the DRG compared with 7.1% of patients without an ulcer. Valued at an average cost per occupied bed-day, the excess cost per case would be between 1,633€ and 2,074€ (26 and 62% above the average reimbursement for these cases). Excess length of stay is not the only source of cost to the hospital. When a hospital-acquired ulcer develops complications, the G-DRG code will only rarely be reclassified to reflect the additional costs. Similarly, where a patient discharged with a pressure ulcer is readmitted within 30 days for treatment of the ulcer, the hospital will not normally receive an additional payment because reimbursement is subsumed within the original episode.

Estimates of the ulcer-associated excess length of stay derived from German national data are consistent with estimates from other studies [12]. In a sample of more than 27 million hospital admissions of patients ≥18 years in the United States in 2006, the excess length of stay for hospitalisations principally for the treatment of pressure ulcers was 9.1 days, and for hospitalisations with pressure ulcer as a secondary diagnosis was 7.7 days [13]. A cross-sectional study of medical records of all inpatients treated in 273 English hospitals between 2005/06 and 2009/10 analysed length of stay and excess bed-days for six patient-safety incidents (death in low-mortality DRGs, pressure ulcer, central line infection, postoperative hip fracture, deep vein thrombosis/pulmonary embolism and postoperative sepsis) [14]. The highest excess mortality risk and excess bed-days were associated with hospital-acquired pressure ulcers. The excess mortality risk was 15.4%, and excess bed-days 15.5 days per admission (95% CI 14.9–16.2).

It is not possible from observational data alone to know the direction of a causal link between the presence of a pressure ulcer and length of stay, because it is possible that patients with longer length of stay are more at risk of developing an ulcer. Several studies have tested for the presence of an independent effect. Graves [15] selected a random sample of 2,000 patients admitted to an acute hospital in Australia between October 2002 and January 2003. Regression methods were used to assess risk factors for excess length of stay. Having a pressure ulcer resulted in a median excess length of stay of 4.31 days (95% CI 1.85–6.78), and pressure ulceration was a statistically significant risk factor (*p* < 0.005). A retrospective observational study analysed data on 3,198 patients aged ≥75 years admitted to University Hospital Aachen in 2008 and 2009 [16]. Patients with a pressure ulcer had a mean excess length of stay of 2.6 days. Several variables had a statistically significant effect on length of stay in a regression model: age, type of admission, ICU stay, ventilation >24 h, infection with multi-resistant pathogens, death during hospitalisation and pressure ulceration (*p* = 0.0011).

Estimates of the incidence of hospital-acquired pressure ulcers among hospitalised patients vary widely. German hospitals have an obligation to report on a series of quality indicators, including the number of hospital-acquired pressure ulcers at stage 2–4 [4]. In 2020, 59,661 hospital-acquired pressure ulcers at stage 2–4 were recorded in a population of 15.043 million inpatient admissions aged ≥20 years, a rate of 0.40% [4]. Adjusting these figures to include stage 1 ulcers at 16.9% (Table 2), the overall recorded incidence was approximately 0.48%. Because hospitals have no incentive to report hospital-acquired ulcers, this may be an underestimate. For example, Lahmann et al. [17] reported a direct comparison of pressure ulcer incidence estimates in German hospitals from two different nationwide multicentre studies. The Kinexus™ PS survey involved observation of patients in participating hospitals by a trained study team, with data recorded on a standardised survey instrument. Pressure ulcer prevalence was recorded on the day of the study, and the incidence of new ulcers was estimated by repeating the observation 1 week later. In 2007 German hospitals were required to report pressure ulcer incidence to the Einrichtungsübergreifenden Qualitätssiche-Rung (EQS), as part of a federal quality assurance initiative. Pressure ulcer status was reported at admission and at discharge to estimate the incidence of hospital-acquired ulcers. Combining data from 2007 and 2008, the incidence of stage 1–4 pressure ulcers in the EQS report was 1.3% compared with 6.7% reported in the Kinexus PS survey [17]. The difference was statistically significant.

Estimates based on observation of patients by trained nurses are likely to be more accurate than statutory reporting. The interdisciplinary decubitus project at Essen University Clinics has been ongoing since 2002 [18]. Pressure ulcer prevalence and incidence were obtained as part of a process in which all inpatients in randomly selected wards were physically assessed by specially trained nurses. The mean age of patients included in the surveys in the 4 years 2003/4 to 2006/7 was 49 years. In this period the incidence of hospital-acquired pressure ulcers ranged from 0.56 to 0.65%. In another study, a database analysis was carried out for all inpatient admissions to University Hospital Dresden between January 2007 and December 2011 [19]. Patients were screened by specialist nurses at regular intervals to assess skin condition. The incidence of hospital-acquired pressure ulcers in the all ages population was 0.78%.

Incidence rates are heavily influenced by the age profile of the surveyed population. The Dresden study also analysed incidence rates by age and by different departments in the hospital. In the age group ≥65 incidence was 1.43% compared with 0.78% overall. In the group aged 80–89 incidence was 2.24, and 6.25% age ≥90 years. The average incidence for medical departments was 1.42%, for surgical specialities it was 2.76%. The incidence among patients in intensive care was 4.78% [19]. A similar study analysed data collected as part of a routine surveillance programme at University Hospital Aachen in 2008 and 2009 [16]. The incidence of hospital-acquired pressure ulcers was 3.4% in an inpatient population aged ≥75 years (mean age 81.6 years). Charité University Hospital, Berlin has been supporting routine pressure ulcer surveillance in a sample of nursing homes and hospitals throughout Germany since 2001. On the day of the survey trained nurses assess patients according to a standardised survey instrument. In 2015 seven hospitals took part in the survey covering 1,133 inpatients. The mean age of included patients was 70 years. The incidence of hospital-acquired ulcers was 2% for ulcers stage 2–4 and 1.4% for stage 1: 3.4% overall [20].

It makes sense to focus preventive strategies on patients with the highest risk, and the evidence from our study suggests that patients aged ≥65 have a higher incidence rate and a longer excess length of stay than other patients. According to the IQTIG quality indicators report for 2020 [4] the most common anatomical locations for incident ulcers were the sacrum (40.19% of the total) and heels (21.87%). Focussing screening resources on these anatomies, older age groups and other patient groups with a similar risk profile is likely to be an effective strategy. For example, for every 5,000 inpatients admitted annually in the ≥65 group, a hospital might conservatively expect between 100 and 150 to develop a new ulcer (2–3%). At an average excess length of stay of 2.5 days, 100–150 new ulcers represent between 250 and 375 bed-days. Valued at an average cost per occupied bed-day (864€), the cost to the hospital would be between 216,000€ and 324,000€ annually. More realistically, saving some of the excess bed-days by preventing pressure ulcers could be used to generate revenue by increasing throughput. The average length of stay in German hospitals in 2020 was 7.2 days [8], and 250–375 bed-days could release sufficient capacity to treat up to an additional 52 cases annually. Assuming an average reimbursement of 3,000€ per case (Table 4), the revenue potential would be up to 156,000€. Even a small reduction in incidence could have an important impact on resource efficiency. Although the focus here is on the impact of pressure ulcers on a hospital, in 2020 almost 87% of pressure ulcers in hospitalised patients were present on discharge [4], and this highlights the fact that most of the resource and cost impact of hospital-acquired ulcers falls on community-based healthcare facilities.

Our study has generated important new data on the impact of pressure ulceration on hospital length of stay without the need for a costly or time-consuming prospective study. Nonetheless, important limitations of this study are the fact that it is not possible from the InEK data to separate ulcers which were hospital-acquired from all the cases with pressure ulcer as a secondary diagnosis. In our analysis we have assumed that the length of stay, and stage distribution characteristics of incident ulcers are the same as for other pressure ulcers recorded as a secondary diagnosis. Because InEK data are presented in aggregated form it is not possible to estimate confidence intervals around differences in length of stay or to test for the statistical significance of observed differences.

## 5 Conclusion

Our evidence shows that under the German DRG system the resource impact of hospital-acquired pressure ulcers is important, and this impact is likely to be underestimated because additional costs associated with readmission, outpatient visits and/or the need for surgical intervention have not been included. Pressure ulcer prevention activities often have a low priority for senior management because the true costs of incident ulcers are not visible. This analysis highlights the value of effective prevention, both for patient welfare and for efficiency in the use of hospital resources.
